# Technology Adoption in Later Life: Cross-Cultural Insights from Hong Kong, Czechia, and Germany

**DOI:** 10.1093/geroni/igaf122.4318

**Published:** 2025-12-31

**Authors:** Nia Yuqing Wang, Yiwen Wu, Josefína Králová, Merle Scheipers, Jaroslava Hasmanová Marhánková, Klaus Rothermund, Helene Fung

**Affiliations:** The Chinese University of Hong Kong, Hong Kong, China (People’s Republic); The Chinese University of Hong Kong, Hong Kong, China (People’s Republic); Charles University, Prague, Hlavni mesto Praha, Czech Republic; Friedrich Schiller University Jena, Jena, Thuringen, Germany; Charles University, Prague, Hlavni mesto Praha, Czech Republic; Friedrich Schiller University Jena, Jena, Thuringen, Germany; The Chinese University of Hong Kong, Hong Kong, China (People’s Republic)

## Abstract

**Background:**

Despite technology’s potential to enhance late-life well-being, older adults exhibit low adoption rates. While the Unified Theory of Acceptance and Use of Technology (UTAUT) identifies predictors of technology adoption among older adults, cross-cultural comparisons remain scarce. This study aims to fill in this gap.

**Methods:**

Semi-structured interviews with 78 adults (aged 70–95 years) across Hong Kong (n = 30), Germany (n = 29), and Czech Republic (n = 19) were conducted. Thematic analysis was done. Independent coders also rated attitudes, intention and actual usage.

**Results:**

Eight themes emerged in explaining technology adoption among older adults: functionality, readiness to adopt, social influence, effort, support, cost, psychological factors, and physical limitations. While Germans and Czechs emphasized readiness to adopt technology, Hong Kongese prioritized functionality and social influence. Smartphones were generally viewed positively across all cultures, with Czechs showing the most favorable attitudes, Germans the least, and Hong Kongese in between. In contrast, technologies that support independent living had low usage across all cultures. Despite this, Czechs showed the highest willingness to adopt, while Germans and Hong Kongese were more hesitant—Germans being the most negative. Finally, common motivators and barriers emerged across all cultures: the primary motivator for use is functionality (e.g., “It’s useful”), while the primary barrier to use is low readiness (e.g., “I don’t want to”). Discussion and

**Conclusion:**

Findings suggest that similar factors motivate technology adaption among older adults across cultures, but cultures shape their priority.

